# Pathway Analysis Based on Attractor and Cross Talk in Colon Cancer

**DOI:** 10.1155/2016/2619828

**Published:** 2016-09-25

**Authors:** Yanxia Liu, Lin Wang, Bingping Wang, Meng Yue, Yufeng Cheng

**Affiliations:** ^1^Department of Radiation Oncology, Qilu Hospital, Shandong University, Jinan, Shandong 250012, China; ^2^Department of Oncology, Shengli Oil Central Hospital, Dongying, Shandong 257034, China; ^3^Department of Gastroenterology, Jinan Central Hospital, Shandong University, Shandong 250013, China

## Abstract

Colon cancer is the third and second most common cancer form in men and women worldwide. It is generally accepted that colon cancer mainly results from diet. The aim of this study was to identify core pathways which elucidated the molecular mechanisms in colon cancer. The microarray data of E-GEOD-44861 was downloaded from ArrayExpress database. All human pathways were obtained from Kyoto Encyclopedia of Genes and Genomes database. In total, 135 differential expressed genes (DEG) were identified using Linear Models for Microarray Data package. Differential pathways were identified with the method of attractor after overlapping with DEG. Pathway cross talk network (PCN) was constructed by combining protein-protein interactions and differential pathways. Cross talks of all pathways were obtained in PCN. There were 65 pathways with RankProd (RP) values < 0.05 and 16 pathways with Impact Factors (IF) values > 100. Five pathways were satisfied with *P* value < 0.05, RP values < 0.05, and IF values > 100, which were considered to be the most important pathways in colon cancer. In conclusion, the five pathways were identified in the center status of colon cancer, which may contribute to understanding the mechanism and development of colon cancer.

## 1. Introduction

Colon and rectal cancer are the third most common forms of cancer in the United States [1]. Colon cancer is the third and second most common cancer form in men and women worldwide [1], causing appropriate 640 thousand deaths each year. It is commonly known as colorectal cancer or large bowel cancer. It is generally accepted that colon cancer mainly resulted from diet in one way or another [2]. Besides, it is also correlated with genetic factors, such as family history of colorectal cancer, and familial adenomatous polyposis [3]. Also, old age [4], gender [5], and presence of adenomatous polyps [6] are risk factors related to colon cancer. Recently, a SNP, rs5995355, in NCF4 was found significantly associated with risk of colorectal cancer after adjustment for both potential confounders and multiple comparisons, but the change of expression was not found in either tumor or normal tissue [7]. Thus, elucidating the molecular mechanisms is critical to clinical diagnosis and treatment for colon cancer. Our purpose of this study is to explore important pathways that reflected mechanism of the occurrence and development of colon cancer by screening differential expressed genes (DEGs) between colon cancer tissues and normal tissues and analyzing the pathways using biological information.

Modern molecular biology indicates that selective expression of genes controls the regulating mechanism in the biology. DEG that has significant difference at expression level between cancer tissues and normal tissues could conduce to analyzing cancer mechanism. Altered pathways between cancer tissue and normal tissue may help to understand the disease status and suggest anticancer therapies. Based on microarray data and Kyoto Encyclopedia of Genes and Genomes (KEGG) database, numerous researches have analyzed biological processes with genes and pathways by using a variety of statistical analysis strategies [8–10].

Attractor is an analytical approach for identifying and annotating the gene sets that best discriminate between cell phenotypes [11]. It can identify core pathways which best contrasted the cell types of interest. With this method, differential pathways between cancer group and normal group can be identified.

Protein-protein interactions (PPIs) provide valuable information about how genes perform functions. Network-based methods have been applied to gain insight into the mechanism from the interaction data [12]. The pathways overlapped with interactional genes are also considered to interact with each other, known as cross talk. Pathways can affect each other through cross talk, rather than working along. Cross talk is valuable in understanding disease, especially cancers, and may play an important role in the invasion and proliferation of cancer cells [13]. Pathway cross talk network (PCN), constructed with pathways and protein interactions according to Li et al. which was first developed to search for colorectal cancer progression and metastasis based on transcriptional data, can be utilized to analyze genome-wide expression profiling data by analyzing how pathways affect each other and the difference between clusters cross talk [14]. But the results were not sufficient since they did not present pathway aberrance. By combining differential pathway analysis and PCN, the analysis can be used for pathways that not only are significantly altered but also influence other pathways. And this has been applied in breast cancer to analyze pathways conducted by Sun et al. [15].

In this study, we tried to explore colon cancer mechanism by analyzing pathways which not only were dysregulated in colon tissues when compared with normal group but also interacted with other pathways. To achieve this goal, gene expression profiles were downloaded from ArrayExpress database to detect differential expressed genes. Human-related pathways were downloaded from KEGG database and PPIs were downloaded from search tool for the retrieval of interacting genes/proteins (STRING) database to identify differential pathways and construct PCN.

## 2. Materials and Methods

### 2.1. Gene Expression Data

#### 2.1.1. Data Resource

Microarray data of E-GEOD-44861 [7], along with its annotation file, was downloaded from ArrayExpress database. There were 56 colon tumor tissues and 55 adjacent noncancerous tissues. The platform in this study was A-AFFY-113-Affymetrix GeneChip HT Human Genome U133A HT_HG-U133A, and the title was “Affymetrix expression data from colon cancer patient tissues.”

#### 2.1.2. Gene Expression Data Preprocessing

Microarray expression data should be preprocessed because they are measured as intensities. Linear Models for Microarray Data (LIMMA) package was chosen to reprocess data with the function of expresso [16]. And the background was corrected with robust multichip average (RMA) [17]. Normalization was performed with quantiles function. Then, we used MAS for corrected perfect match (PM)/mismatch (MM) [18]. Medianpolish function was used for summarizing expression data. After probe filtration with featureFilter function, 12493 genes were obtained.

#### 2.1.3. Differential Expressed Genes (DEGs) Screening

DEGs have become an important method in studying tumor-related genes. They contribute to illuminating mechanism of a tumor. In this study, LIMMA method was applied to screen DEG. The values of |log FC| ≥ 1.5 and *P* value ≤ 0.01 were selected as the cut-off criteria.

### 2.2. Pathway Data

The common databases regarding biology are Kyoto Encyclopedia of Genes and Genomes (KEGG) database (http://www.kegg.jp/) [19] and Reactome database (http://www.reactome.org/) [20]. In this study, we downloaded pathways from KEGG database. After deleting the pathways containing no genes, 294 pathways were obtained.

### 2.3. Protein-Protein Interaction (PPI) Data

PPI data has become an important source of protein function and relationship information in microbiology, molecular biology, computational biology, and medicine. And it can provide valuable information regarding how genes carry out their biological functions [14]. The PPI data can be downloaded from search tool for the retrieval of interacting genes/proteins (STRING) database [21]. In total, 787896 PPIs were obtained.

### 2.4. Differential Pathways Analysis

To screen differential pathways, attractor method was applied [11].

Genes in normal group and disease group were treated with KEGG enrichment analysis. Attractors were obtained with GSEA-ANOVA, an analysis of variance-based implementation of a gene set enrichment algorithm.

From this model, *F*-statistic of gene was figured out with

(1) $$\begin{array}{c} F^{\left(i\right)}=\frac{{MSS}_{i}}{{RSS}_{i}}, \end{array}$$

where MSS_*i*_ denotes the mean treatment sum of squares

(2) $$\begin{array}{c} {MSS}_{i}=\frac{1}{K-1}\overset{K}{\underset{k=1}{\sum }}r_{k}{\left[\;y_{\cdot k}^{\left(i\right)}-y_{\cdot \cdot }^{\left(i\right)}\;\right]}^{2} \end{array}$$

and RSS_*i*_ denotes the residual sum of squares

(3) $$\begin{array}{c} {RSS}_{i}=\frac{1}{N-K}\overset{K}{\underset{k=1}{\sum }}\;\overset{r_{j}}{\underset{j=1}{\sum }}{\left[\;y_{jk}^{\left(i\right)}-y_{\cdot \cdot }^{\left(i\right)}\;\right]}^{2}. \end{array}$$

For pathway *p* consisting of *g*_*p*_ genes, the *T*-statistic takes the following form:

(4) $$\begin{array}{c} T_{p}=\frac{\left[\left(1/g_{p}\right)\sum_{i=1}^{g_{p}}F^{\left(i\right)}\right]-\left[\left(1/G\right)\sum_{j=1}^{G}F^{\left(i\right)}\right]}{\sqrt{\left({S_{p}}^{2}/g_{p}\right)+\left({S_{G}}^{2}/G\right)}}, \end{array}$$

where *G* denotes the total number of genes in a pathway and *S*_*p*_^2^ and *S*_*G*_^2^ were defined as sample aberrances.

After performing *T*-test and adjusting by false discovery rate (FDR) of Benjamini-Hochberg [22], *P* values of each pathway were obtained.

### 2.5. Construction of Pathway Cross Talk Network (PCN)

To analyze interactions between pathways, a PCN was constructed as described by Li et al. [14].

#### 2.5.1. Interactions

It is assumed that cancer-associated gene's dysregulation of expression can lead to the differential expression of its interacting genes when there is no network rewiring. We used gene expression correlation to measure the dynamic action of the PPIs.

In both disease group and background group, Spearman correlation coefficient of each PPI was calculated with the following formula [23]:

(5) $$\begin{array}{c} r_{E_{ij}}=\frac{\sum_{k}\left(x_{ik}-{\overset{-}{x}}_{i}\right)\left(x_{jk}-{\overset{-}{x}}_{j}\right)}{\sqrt{\sum_{k}\left(x_{ik}-{\overset{-}{x}}_{i}\right)\sum_{k}\left(x_{jk}-{\overset{-}{x}}_{j}\right)}}, \end{array}$$

where *E*_*ij*_ is the PPI between gene *V*_*i*_ and gene *V*_*j*_; *k* is the *k*th sample; *x*_*jk*_ is the rank of *V*_*i*_ of *k*th sample; *x*_*ik*_ is the rank of *V*_*j*_ of *k*th sample; ${\overset{-}{x}}_{i}$ and ${\overset{-}{x}}_{j}$ are the average ranks of *V*_*i*_ and *V*_*j*_ in the samples, respectively:

(6) rEij−=12rEij1+rEij2,ΔrEij=rEij1−rEij2,

where *r*_*E*_*ij*1__and *r*_*E*_*ij*2__ represent the Spearman coefficients of *E*_*ij*_ in compared samples, respectively.

The gene pairs are considered to interact intensively if |Δ*r*_*E*_*ij*__| ≥ 0.5. If |Δ*r*_*E*_*ij*__| < 0.5 but the two genes belong to differential expressed genes, they are also considered to have strong interactions and should be reserved.

#### 2.5.2. Weight

Weight represents the number of PPI in the network [24]. Only gene pairs with weight > 5 which are considered to have strong interactions are recorded and used to construct a disease-related PCN.

#### 2.5.3. Degree

By analysis of the topological characteristics in the network, all node degrees were obtained. The degree ratio was defined as the degree of a node in disease-related PCN to that in background PCN.

We introduced a concept of pathway score to examine the pathway status in the disease. The formula was shown as

(7) $$\begin{array}{c} Pathway\;\;score=\frac{Degree\;\;in\;\;disease}{Degree\;\;in\;\;normal}. \end{array}$$

### 2.6. Comprehensive Analysis of Pathways

For a comprehensive analysis of disease-related genes and pathways, we introduced nonparametric rank product (RankProd) approach [25] to find important pathways. The formula was shown as

(8) $$\begin{array}{c} RP=\left(\;\frac{Rank\;\;inter}{Total}\;\right)\times \left(\;\frac{Rank\;\;outer}{Total}\;\right), \end{array}$$

where inter indicated attracting *P* value of a pathway and outer indicated degree of a pathway.

An impact factor (IF) concept was also introduced to examine the significance of a pathway, which was shown as

(9) $$\begin{array}{c} IF=outer\times \left(\;1-inter\;\right). \end{array}$$

## 3. Results

### 3.1. DEG Analysis

Based on the microarray data of E-GEOD-44861, 135 DEGs between colon cancer tissues and normal tissues were screened out with |log FC| ≥ 1.5 and *P* value ≤ 0.01 by using the method of LIMMA.

### 3.2. Differential Pathways Analysis

Compared with normal group, a total of 18 differential pathways with *P* value < 0.05 were obtained in the cancer group as shown in Table 1. These pathways were colon cancer-related pathways, such as “bladder cancer” pathway and “neuroactive ligand-receptor interaction” pathway, and five pathways were correlated with metabolism. The top nine ranked pathway with significant levels (*P* value < 0.01) were identified, which were significantly different between normal group and cancer group.

### 3.3. Pathway Cross Talk Network Analysis

Pathways and protein interactions were integrated to a global PCN. In the network, nodes represented as pathways and edges denoted cross talk between pathways. We analyzed node degrees of the network, which indicated the connections among pathways. Cross talks in background were shown in Supplemental Data 1 (in Supplementary Material available online at http://dx.doi.org/10.1155/2016/2619828) and cross talks test groups were shown in Supplemental Data 2.

Pathways owning more connections with others indicate that they are more important in the network. In the cancer-related PCN, the pathways with large degree indicated they were more important in the case of cancer.

Degree ratio of node degree in test group to that in normal group, named as pathway score, was computed and the scores were ranged from 0 to 0.7. There were 80 pathways with degree ratios < 0.01, of which 58 pathways were with degree ratios = 0, which indicated that the pathway connections with others were vanished when people were suffering from colon cancer. The important pathways were those with higher pathway scores. Top ten ranked pathways with pathway scores and pathway degree were listed in Table 2.

**Figure 1:**
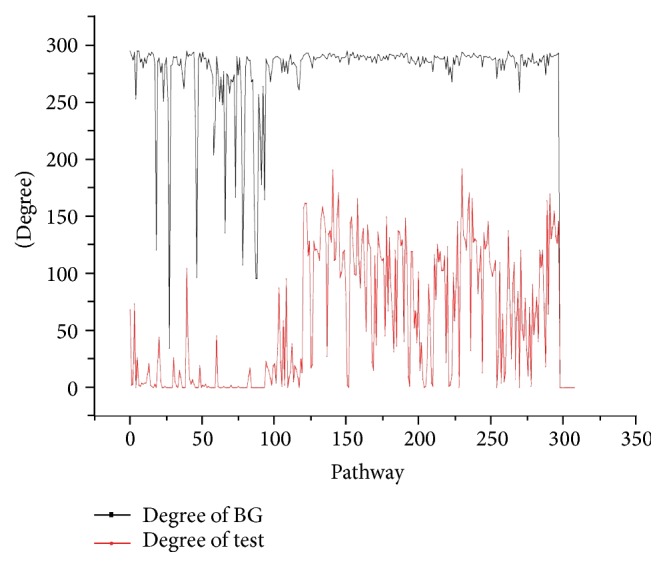
Degrees of pathways in background group (BG) and test group. Red color indicates test group, and black color indicates BG.

Degrees of pathways were compared between test group and BG group, as shown in Figure 1. Degrees of pathways in test group were much less than degrees of pathways in BG group.

### 3.4. Comprehensive Analysis of Pathways

There were two definitions that illuminate the status of a pathway in colon cancer, RP value, and IF value.

**Figure 2:**
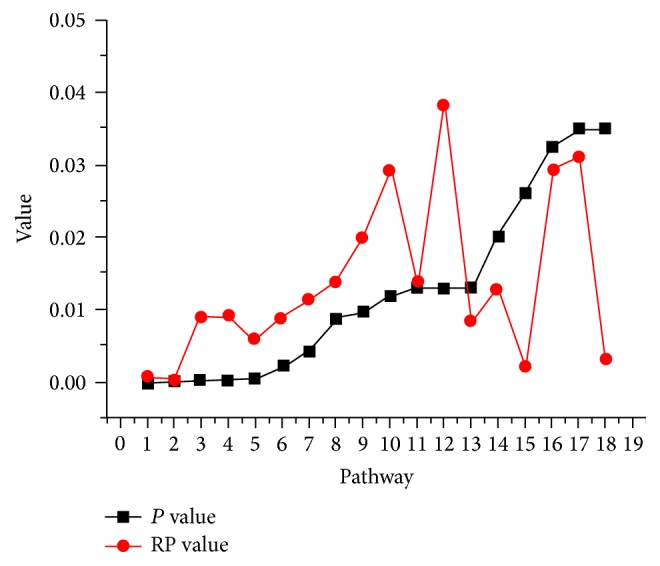
RankProd (RP) values of the 18 pathways with *P* values < 0.05. Color in red indicates RP value, and color in black indicates *P* values.

According to the ranks of degree and *P* value in a pathway, RP value was computed, ranging from 0 to 1. There were 65 pathways with RP values < 0.05. On the basis of *P* value < 0.05, 18 pathways were eligible, as shown in Figure 2, and they were considered to be important pathways.

**Figure 3:**
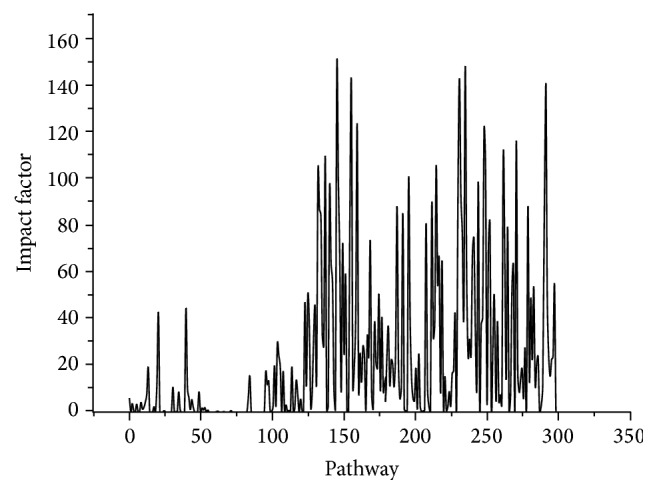
Impact factor (IF) values of the 300 pathways.

IF value indicates the significance of a pathway that performs in colon cancer.  Figure 3 displayed all the IF values of pathways. There were 16 pathways with IF values > 100, which were considered to be important pathways. The top five ranked pathways with large values were “neuroactive ligand-receptor interaction” pathway, “microRNAs in cancer” pathway, “pathways in cancer” pathway, “cell cycle” pathway, and “human T-cell leukemia virus (HTLV-I) infection” pathway.

After comparing pathways with RP values, *P* values, and IF values, five pathways were obtained which were identified important in all three factors: “bladder cancer” pathway, “alcoholism” pathway, “dopaminergic synapse” pathway, “microRNAs in cancer” pathway, and “cell cycle” pathway.

## 4. Discussion

Due to the fast development of bioinformatics, network-based approaches have become increasingly important to search for cancer mechanisms [26], such as coexpression network and PPI. PCN, based on pathways and PPI, plays a key role in identifying important pathways. To explore mechanism of colon cancer, PCN was applied to search for core pathways.

The microarray data of E-GEOD-44861 was selected to explore mechanism of colon cancer, since it was a representative study in recent years. In the study of Ryan et al. [7], the focus point was NADPH-related pathways, while, in this study, we mainly focused on searching for core dysregulated pathways in colon cancer from all human-related pathways.

With gene expression profiles of 56 cancer tissues and 55 normal adjacent tissues, 18 differential pathways were identified by attractor procedure. These pathways were greatly changed in cancer tissues compared with normal tissues.

As pathways function with each other and do not work alone, cross talk in pathways is needed for analysis. In the pathway-based PCN, degree indicates connections between pathways. RankProd method was applied to rank pathways in degree and *P* value, generating two factors, RP value and impact factor. After comprehensive analyses of *P* value, RP value, and IF values, we identified 5 pathways as important pathways. They were not only with significant changes between cancer and normal tissues but also with many connections with other pathways. Once 1 of the 5 pathways changed, pathways connected with it will be influenced.

“Bladder cancer” pathway was identified to be associated with colon cancer. This pathway mainly participated in urothelial carcinoma. Urothelial tumors arise and evolve through divergent phenotypic pathways. Some tumors progress from urothelial hyperplasia to low-grade noninvasive superficial papillary tumors. More aggressive variants either arise from flat, high-grade carcinoma in situ (CIS) and progress to invasive tumors or arise as invasive tumors. Low-grade papillary tumors frequently show a constitutive activation of the receptor tyrosine kinase-Ras pathway, exhibiting activating mutations in the HRAS and fibroblast growth factor receptor 3 (FGFR3) genes. In contrast, CIS and invasive tumors frequently show alterations in the TP53 and RB genes and pathways. Invasion and metastases are promoted by several factors that alter the tumor microenvironment, including the aberrant expression of E-cadherins (E-cad), matrix metalloproteinases (MMPs), and angiogenic factors such as vascular endothelial growth factor (VEGF) [27].

“Alcoholism” pathway is a chronic relapsing disorder related pathway. Alcoholism is progressive and has serious detrimental health outcomes. As one of the primary mediators of the rewarding effects of alcohol, dopaminergic ventral tegmental area (VTA) projections to the nucleus accumbens (NAc) have been identified. Acute exposure to alcohol stimulates dopamine release into the NAc, which activates D1 receptors, stimulating PKA signaling and subsequent CREB-mediated gene expression, whereas chronic alcohol exposure leads to an adaptive downregulation of this pathway, in particular, of CREB function. The decreased CREB function in the NAc may promote the intake of drugs of abuse to achieve an increase in reward and thus may be involved in the regulation of positive affective states of addiction. PKA signaling also affects NMDA receptor activity and may play an important role in neuroadaptation in response to chronic alcohol exposure [28].

“Dopaminergic synapse” pathway is correlated with nervous system. Dopamine (DA) is an important and prototypical slow neurotransmitter in the mammalian brain, where it controls a variety of functions including locomotor activity, motivation and reward, learning and memory, and endocrine regulation. Once released from presynaptic axonal terminals, DA interacts with at least five receptor subtypes in the central nervous system (CNS). Through diverse cAMP- and Ca^2+^-dependent and Ca^2+^-independent mechanisms, DA influences neuronal activity, synaptic plasticity, and behavior. Presynaptically localized D2Rs regulate synthesis and release of DA as the main autoreceptor of the dopaminergic system [29, 30].

“MicroRNAs in cancer” pathway is involved in a cluster of small nonencoding RNA molecules of 21–23 nucleotides in length, which controls gene expression posttranscriptionally via either the degradation of target mRNAs or the inhibition of protein translation. Using high-throughput profiling, dysregulation of miRNAs has been widely observed in different stages of cancer. The upregulation of specific miRNAs could result in the repression of tumor suppressor gene expression, and conversely the downregulation of specific miRNAs could lead to an increase of oncogene expression; both these situations result in tumor growth and progress. The miRNA signatures of cancer observed in various studies differ significantly. These inconsistencies result from the differences in the study populations and methodologies [31, 32].

“Cell cycle” pathway functions in response to DNA damage by activating signaling pathways that promote cell cycle arrest and DNA repair. When responding to DNA damage, the checkpoint kinase ATM phosphorylates and activates Chk2, which in turn directly phosphorylates and activates p53 tumor suppressor protein. p53 and its transcriptional targets play an important role in both G1 and G2 checkpoints [33]. ATR-Chk1-mediated protein degradation of Cdc25A protein phosphatase is also a mechanism conferring intra-S-phase checkpoint activation [34].

Besides, some identified pathways were involved in the regulation of NADPH, such as “fatty acid metabolism” and “oxidative phosphorylation,” which was similar to the results of Ryan et al. [7].

Still, there are limitations in the paper. The results were generated from bioinformatics analysis, which still need clinical data to be verified.

## 5. Conclusion

In this study, we concentrated on exploring important pathways that reflected mechanism of the occurrence and development of colon cancer. We identified DEG and differential pathways by comparing colon cancer tissues and normal tissues. After comparing PCN between cancer and normal, we identified 5 important pathways, which may give new insights into the underlying biological mechanisms driving the progression of colon cancer and should be paid close attention to in further research.

## Supplementary Material

The crosstalks between pathways were presented in count list.

## Figures and Tables

**Table 1:** Differential pathways with *P* value < 0.05.

KEGG ID	Term	*P* value
05219	Bladder cancer	2.23*E* − 05
04080	Neuroactive ligand-receptor interaction	7.08*E* − 05
00071	Fatty acid degradation	0.000163
04740	Olfactory transduction	0.000239
04932	Nonalcoholic fatty liver disease (NAFLD)	0.000372
00190	Oxidative phosphorylation	0.002173
05322	Systemic lupus erythematosus	0.004022
03010	Ribosome	0.008739
01212	Fatty acid metabolism	0.009654
00920	Sulfur metabolism	0.011812
05012	Parkinson's disease	0.012836
05033	Nicotine addiction	0.012936
05034	Alcoholism	0.012936
04728	Dopaminergic synapse	0.020141
05206	MicroRNAs in cancer	0.025966
00860	Porphyrin and chlorophyll metabolism	0.032464
00520	Amino sugar and nucleotide sugar metabolism	0.035002
04110	Cell cycle	0.035002

**Table 2:** Top ten pathways ranked by pathway scores.

Pathways	BG degree	Test degree	Pathway score
PI3K-Akt signaling pathway	291	192	0.659794
Pathways in cancer	293	193	0.658703
Cytokine-cytokine receptor interaction	289	172	0.595156
Proteoglycans in cancer	290	172	0.593103
HTLV-I infection	292	171	0.585616
Viral carcinogenesis	289	167	0.577855
Focal adhesion	291	167	0.573883
Tuberculosis	291	165	0.567010
Rap1 signaling pathway	291	163	0.560137
MAPK signaling pathway	292	163	0.558219

BG: background, pathways in normal group; degree ratio: the ratio of degree in test group to that in BG group; HTLV: human T-cell leukemia virus; MAPK: mitogen-activated protein kinase.
